# Stigma and labelling of immigrant personal support workers during COVID-19 pandemic in Ontario: Implications for policy

**DOI:** 10.1371/journal.pone.0340589

**Published:** 2026-01-08

**Authors:** Quinn Talbot, Evans Batung, Isma Yusuf, Francisca Omorodion, Godwin Arku, Isaac Luginaah

**Affiliations:** 1 Department of Geography and Environment, Western University, London, Ontario, Canada; 2 Department of Sociology and Criminology, University of Windsor, Windsor, Ontario, Canada; University of Auckland, NEW ZEALAND

## Abstract

The COVID-19 pandemic had a profound impact on the livelihoods of people from diverse socio-economic backgrounds in Canada. In this study, we focus on immigrant essential workers, who played pivotal roles in sustaining Canada’s socio-economic and healthcare sectors during this crisis. Drawing data from twenty-five in-depth interviews with immigrant personal support workers, we found that immigrant personal support workers experience a complex and compounding type of stigma, traceable to their location at race, gender, class, and ethno-national lines. Using intersectionality and stigma as guiding theoretical frameworks, our study looks at the impact of stigma and labelling on personal support workers in the Windsor, Ontario, Canada, the intersectional character of such stigma, and the novel challenges introduced by the pandemic. The findings show that although some people praised personal support workers for their work on the frontlines, since others viewed them as carriers of the COVID-19 virus, certain changes had to be made to their daily routines to avoid being seen as a personal support worker. Additionally, challenges in the workplace included lack of personal protective equipment and unsafe work environments. Immigrant personal support workers were affectionately labelled ‘essential’ workers, as they were performing critical tasks at the forefront of the pandemic response. Personal support workers also found themselves in a duality of public opinion by being recognized as ‘essential’ workers, yet being simultaneously stigmatized as potential sources for the spread of COVID-19. The findings provide insights into changes that need to be made in the future toward improving immigrant personal support workers’ health and well-being in times of crises.

## Introduction

During the COVID-19 pandemic, it became indisputable that ‘low-skilled’, ‘low-paying’ workers were indispensable to both the broader provision of health care and the Canadian economy at large. These individuals belong to the wider professional category of ‘front-line workers’ – the individuals who take on the risky role of interacting with, servicing, and providing direct care to customers, clients, patients, and residents. While what exactly constitutes ‘front-line’ work ranges greatly from social/health care provision and cleaning to food processing/distribution and hospitality, the category becomes significantly less broad once we focus specifically on who exactly makes up the population of *front-line healthcare workers* [1,2]. According to Statistics Canada [1], over one-third (approximately 34%) of essential service workers were immigrants, compared to just over one-fifth (21%) in other sectors.

Due to their frontline duties, personal support workers (PSWs) and other healthcare workers (HCWs) were affectionately ascribed the label of ‘essential worker’ by the Canadian public, as they fulfill a necessary role that falls outside of the stay-at-home and lockdown orders [3,4]. The importance of this profession was, in fact, underscored by Federal Minister of Immigration, Refugees and Citizenship Canada (IRCC), Marco Mendicino, who asserted that “immigrants, temporary foreign workers and international students are making important contributions as frontline workers in health care and other essential service sectors. We know and value their efforts and sacrifices to keep Canadians healthy and ensure the delivery of critical goods and services” [5]. At the onset of the pandemic when many Canadians were on lockdown – yet able to work remotely from home – it was ‘essential’ workers who put themselves and their families at risk of catching, carrying, and circulating the coronavirus by working in-person.

The contributions of PSWs were also widely highlighted by various media outlets, as they played a crucial role, especially in long-term care (LTC) homes where COVID-19 took a heavy toll [6]. This led to a sudden increase in recognition of their efforts by the general public. Unfortunately, in comparison to other workers, PSWs do not receive adequate pay to compensate for their risky jobs – making $19 per hour on average [7]. In addition to being the lowest paid health workers in Ontario, this profession is highly ethno-racialized *and* gendered, as the majority of PSWs are highly-skilled racialized immigrant women, from across the continents of Africa, Asia, South America, and the Caribbean Islands. Additionally, the pandemic saw PSWs precarious financial location compounded by a multi-pronged form of stigmatization, which carried gendered, ethno-racial, national, and medical connotations. Their overwhelmingly gendered, racialized bodies ‘from elsewhere’ were stigmatized as carriers of COVID-19 virus. Consequently, PSWs found themselves in a duality of public opinion as being recognized as ‘essential’ workers, yet at the same time being stigmatized as potential sources for the spread of COVID-19.

To broaden our understanding of how to respond to such a duality of reactions in the context of future pandemics, the objective of this study is to explore essential immigrant PSWs particular experiences and experiences of stigma prior to and during the pandemic. As we work through the accounts of marginalization at the hands of multiple parties and pathologization, we examine the interwoven racial, gendered, ethno-national, and classed implications that underlies the stigma that they encounter. Drawing upon theories of stigma and intersectionality, we connect these threads to contend that race, gender, class, and nationality fused to create a unique type of stigma experienced by PSWs.

### Overview of personal support workers in Ontario

Personal support work involves providing personal care services to any person in their home or in supported independent living environments such as LTC homes, to meet their supportive, physical and psycho-social needs [8]. Many of these clients tend to be socially isolated and PSWs assist with activities of daily living. [9]. Prior to the pandemic, PSWs lacked recognition for their immense contribution to the healthcare sector, although they have been pivotal in the healthcare system for decades, notably in LTC homes [10]. For instance, PSWs were often viewed negatively by society, as the media often portrayed them as “lacking compassion” [11]. Relative to other frontline HCWs, PSWs lack mandatory standardized training, contributing to PSWs “low-skill” status [12]. Their limited training, however, does not mean that they are themselves unskilled, for a significant number of PSWs are ethno-racialized migrant women who were trained as nurses or physicians in their home countries. Despite being highly skilled, their educational and professional qualifications are not recognized in Canada, creating a scenario where they must take lower skilled – and thus, lower-paying – jobs, often multiple in number to survive within the perilous Canadian economy [13]. The economic precariousness of being a PSW intensified during the pandemic, when the Government of Ontario, in an effort to reduce the transmission of COVID-19, mandated that Ontario-based PSWs were no longer allowed to work at more than one health care facility [14].

Yet, their place in the health system will be increasingly necessary due to the increased care demands arising from an aging population [15]. For instance, it is expected that the proportion of the Canadian population 65 years and older will represent between 24% to 26% of the population by 2061, compared to 14% in 2009 [16].

There are 627 LTC homes in Ontario; of which the provincial government provides funding for all staff and supplies related to personal care in this setting [17]. Of the 100,000 PSWs working in Ontario, over 57,000 work in LTC facilities and 34,000 in home care and community support [18]. PSWs working in home and community care may be fully or partially funded by the Government of Ontario, if the client is considered qualified for these services [19].

In Ontario, PSWs can pay to be part of the Ontario Personal Support Workers Association (OPSWA), a professional association dedicated to advocating for PSWs. While they aim to promote professional standards within the field, they do not have the authority to enforce or maintain standards. Furthermore, because membership in this organization is voluntary, not all PSWs are represented or supported by the OPSWA, resulting in inconsistencies in standards across the profession [9]. Therefore, each employer tends to rely on their own policies to tackle emerging issues. It is within this context we explore the issues confronted by PSWs during the COVID-19 pandemic.

While the Canadian body of COVID-19 research has grown to include studies attentive to PSWs and ‘essential’ workers broadly construed [20,21], it is crucial that dialogical and discursive space is continuously held for the wide category of PSWs, as this group remains discursively marginalized within the health care profession. Studies have addressed experiences of immigrant workers or PSWs separately, however, little attention has been given to those who belong to both categories. This leaves a critical gap in understanding immigrant PSWs’ unique experiences during the pandemic. Attention must be continuously allocated to immigrant PSWs, whose experiences of exclusion are often flattened within health care discourses. Hence, this paper focuses attentively on immigrant PSWs, and the experiences with stigma and labelling that emerged at the intersection of race, gender, class, and ethno-national identity amidst the pandemic.

### Theoretical underpinning

This study is informed by two theoretical concepts: Crenshaw’s [22,23] intersectionality and Goffman’s [24] stigma, both of which fit seamlessly together. Beginning with intersectionality, the concept is grounded in the contention that systems of domination (e.g., race, class, gender, nationality) are not atomized entities, but are interwoven, interdependent structures which compound (i.e., *intersect*) to create a unique, intense, and indivisible experience. Coined by Crenshaw [22] but imbued with roots of extensive Black feminist theory [25–28], intersectionality departs from – and vehemently critiques— ‘single-issue’ approaches to theory, such as the class-centric (see: Marxism), gender-exclusive (first and second wave feminism), and race-foci (early Black liberatory politics) lenses that dominated critical theory in the 20^th^ century. Instead, intersectionality sees such social systems as indelibly linked, with issues of class being inextricable from race, gender, sex, nationality, age; gender indivisible from race, class, nationality; race inseparable from gender, sex, nationality, age – and so on. As intersectional feminist Audre Lorde [29] vocally contended in her “Learning from the 60s” speech, “there is no such thing as a single-issue struggle because we do not live single-issue lives.” Through the prism of intersectionality, social subordination and ostracization can never be read as routable to one form of domination (e.g., racialization/racism; gendering/sexism or misogyny), but always ever charged with another or multiple forms of social exclusion (e.g., racialization/racism *and* gendering/sexism or misogyny). An intersectional lens is thus fruitful for carefully teasing apart the multifaceted complexities of the stigma(s) faced by immigrant PSWs, so as they are shaped by inseparable classed, raced, gendered, and ethno-national identities.

An intersectional lens is pressed alongside, and complemented by, Goffman’s [24] stigma – the leading and most cited sociological work on stigma. For Goffman [24], stigma is a powerful, discrediting, and tainting social identification that fundamentally changes the way individuals’ view themselves or how they are viewed by other people. Stigmatization occurs in both verbal and non-verbal forms that express the devaluation of the target, based on their actual or perceived membership in a stigmatized group [30]. Stigmas are social constructs, meaning they are communicated to and among community members in a manner that the broader public becomes socialized to recognize those who are stigmatized and to enact devaluation [31].

Goffman [24,32] organizes stigmas – sometimes referred to as ‘spoiled identities’ – into three categories: (1) physical deformities; (2) individual ‘moral’ or ‘blemishes’ of character (e.g., mental health illnesses, queer identities, substance addiction, promiscuity, poverty); and (3) tribal stigma (e.g., race, ethno-national origin, religious identity) [32]. As people are societally reduced to one (or more) of the aforementioned spoiled identities, they come to become an individuals’ ‘master status,’ or dominant way in which they are perceived through social interactions within their society [33]. The consequences of bearing a spoiled identity as ‘master status’ has been extensively used in infectious disease literature such as HIV/AIDS and Ebola [34,35]. In the context of the pandemic and PSWs, the categories of individual/moral character of blemishes and tribal stigma are particularly relevant. Within the healthcare system, PSWs are often positioned at the lower end of the occupational hierarchy due to their educational requirements which are considered minimal compared to other healthcare professions [12,15]. This structural positioning may contribute to their work being perceived as less skilled or less prestigious than other healthcare roles. Additionally, the PSW profession carries a form of tribal stigma due to its demographic composition, being predominantly made up of ethno-national (migrant), racialized women. For this reason, intersectionality and stigma work in theoretical tandem, for the stigma negotiated by PSWs carry indivisible racial, ethno-national, and classed connotations, and can be slotted neatly into the categories of individual/moral character blemishes and tribal stigma.

Stigmatization can lead to avoidance, social rejection and dehumanization as people are reduced to stereotypic caricatures that remove them of their individuality and complexity [36]. Often, this reduction into one or more categories of stigmatization functions to fortify those in positions of power, for they benefit from constructing themselves in hierarchal opposition to those identities made ‘spoiled’ [37]. This enables people in power to create and maintain inequalities between groups [38]. Thus, stigma can create divisions between ‘normals’ and ‘abnormals’ [24]. Related to the COVID-19 pandemic, this is a division between those who were working in high-risk occupations including PSWs, and the those who were able to work from home. Furthermore, stigma may lead to intensely skewed social interactions between the stigmatized and non-stigmatized individuals [38].

Many of the core attributes highlighted in the theoretical conceptualization of stigma can be further understood by the concept of stigma power which embodies the mechanics of stigma in the reproduction and distribution of social inequalities [39]. This can be demonstrated by the fact that PSWs continued to work in high-risk conditions for low wages, whereas those in upper-class occupations are able to safely work from home for a higher wage.

Labelling theory proposes that people see themselves as how others see them, which is useful in provoking certain reactions [40]. It has been recognized that people may also be labelled based on the kinds of work they do. Labelling theory therefore suggests that the label that is given to an individual can stimulate them to behave according to the label, and even influence how they behave in the future [41]. Notwithstanding this reverberating capability of labelling, a noteworthy characteristic is that labels are not permanent, and may be modified or removed, making labelling a relational process [40]. It is worth noting that, although labelling can produce positive impacts on individuals and groups, its effects are typically negative, revolving typically around deviance, deficiency, and difference [42]. Under such circumstances, labelling influences social processes whereby certain people are defined as ‘different’ by society or in the case of COVID-19 being labelled as ‘essential’. Yet, according to Sjöström [42], there is always some form of power dynamics when individuals or groups are labelled. Thus, labelling can often be understood as a form of social control with several hidden connotations.

When the pandemic began, PSWs and several other occupations were categorized and similarly labelled ‘essential’, with a potential underlying objective being to motivate. This label led to increased recognition of their efforts by the public, where many viewed PSWs as ‘heroes’. The concept of healthcare ‘heroes’ became increasingly popular across various platforms of media including newspaper articles, news websites, videos, corporate and medical information websites and magazines [43]. The ‘heroes’ label spread quickly through the media and the new concept quickly had an influence on the public, corporations, and healthcare institutions. At the public level, we began to see people showing appreciation through acts such as cheering for uniformed HCWs. At the corporate level, we began to see companies offering ‘healthcare hero discounts’ along with posting signs of appreciation towards the ‘healthcare heroes’. At the healthcare institution level, this led to an abundance of food donations, and the display of banners with statements such as ‘heroes work here’ [44].

In the context of the COVID-19 pandemic, labelling can be viewed in terms of increasing the complexity and interaction between the public, policy makers and professionals. Before the COVID-19 pandemic, the term ‘essential’ worker was rarely used outside of certain areas such as labor economics and union negotiations, where it referred to a narrow range of occupations. However, during the pandemic, the label ‘essential’ worker gained significant recognition and was applied to a broader spectrum of jobs deemed critical to maintaining daily life [45]. This shift in recognition raises questions about the critical interpretation of the importance of such labelling certain workers as ‘essential’, particularly in the context of the post-pandemic world and in preparation for future pandemics.

Labelling would have a significant internal impact on a person and affect their behaviors. People feel like ‘somebody’ when accepted and ‘nobody’ when they are not [46]. Furthermore, most people desire to have a high evaluation of themselves, which involves how others interpret their capacity and achievement. This influences self-esteem, and can lead to one feeling inferior, weak, and hopeless [47]. Labelling theory is therefore important when considering the positionalities of ‘essential’ workers, as they were not labelled ‘essential’ prior to the pandemic, and potentially with a diminished label when the pandemic winds down.

## Methods

The study was conducted through a constructivist lens, which uses qualitative data methods to focus on experiences of participants. Constructivist researchers believe that knowledge is context-specific, meaning that the findings are not generalizable [48]. The group being studied are specifically immigrant PSWs whose experiences during the pandemic may differ from that of Canadian-born PSWs. Additionally, immigrants make up approximately 41.2% of the PSW workforce in Ontario, hence the importance of learning about their experiences as a unique group [49]. Therefore, this research seeks to understand participants’ internalized experiences. Specifically, the purpose of this study was to understand the impact that stigma, labelling and intersectionality had on producing challenges among immigrant PSWs from Windsor, Ontario and surrounding areas. The following research questions guided the study: 1. What role did stigma and labelling have in creating challenges for immigrant PSWs during the pandemic? 2. What role did intersectionality play in the kind of challenges experienced by immigrant PSWs during the COVID-19 pandemic?

### Data collection

Within Windsor, organizations that provide support services to immigrants such as the YMCA and the Cross-Cultural Learning Centres helped recruit participants for the study. These groups helped us to determine communal vantage points that immigrant frequented during the pandemic. Once locations were determined, research posters were posted that shared the study details and scope, key contacts, and disclosures to help guide participants in deciding to participate. We conducted in-depth interviews to collect data on participants’ experiences of stigma and labelling [48]. It made sense to use this method as it allowed participants to share their unique personalized experiences during the pandemic. From October 8^th^ 2020 to March 21 2021, we interviewed twenty-five immigrant PSWs living in Windsor, Ontario (Table 1). The interviews lasted an average of 49 minutes and explored participants’ lived experiences, values, and perspectives on the COVID-19 pandemic. Participants who expressed interest in taking part in the research were briefed about the research and eligibility requirements. Inclusion criteria required participants to be employed as a PSW, have resided in Southwestern Ontario for at least one year, and can speak English. An interview guide was used throughout the interviews and major themes of the interview guide included the impact of COVID-19 on livelihood, the role of an essential frontline working during a pandemic and being labelled as an essential worker. Since interviews were conducted via phone/online due to COVID-19 restrictions, verbal consent was obtained from all individual participants included in the study. Ethics approval for this research was received from the Western University Non-Medical Research Board on August 6^th^ 2020.

**Table 1:** Overview of the participants (n = 25).

Sample Characteristics	Number of Participants
**Gender**	
Female	22
Male	3
**Age**	
21-30	7
31-40	6
41-50	8
50+	4
**Years Lived in Canada**	
1-5	9
6-10	1
11-15	6
16-20	4
20+	5
**Region**	
Africa	14
Asia	6
Caribbean/South America	5
**Higher Level of Education**	
High school	2
College	10
University	13

### Data analysis

With consent from participants, each interview was recorded using a digital audio recorder. The audio clips were then transcribed using Microsoft Word and imported into the NVIVO software. The transcripts were analyzed to determine preliminary themes using inductive analysis – a method that primarily uses detailed readings of raw data to derive concepts, themes, or a model through interpretations made from the raw data by an evaluator or researcher. Once the themes were determined, the transcripts were re-read in-depth multiple times to determine categories and to organize codes into the corresponding categories. The findings in this form of analysis are influenced by the evaluation objectives or the questions asked by the researcher. Furthermore, the findings arise directly from the analysis of raw data, not from priori expectations or models [48]. The use of this form of analysis is appropriate given the study context, as COVID-19 is a relatively new topic, and the findings are subjective.

## Results

In this section, we present results based on the emerging themes on the impact of COVID-19 on immigrant PSWs in Windsor, Ontario. Direct quotations from participants are also provided to help contextualize the findings. The results are presented based on experiences recalled by participants from two different points in time; prior to and during the pandemic. This allows for comparisons for each of the common themes labelling, stigma, and employment and workplace conditions at the different times.

### PSWs perceptions before the pandemic

#### Lack of recognition/appreciation.

Prior to the pandemic, immigrant PSWs were stigmatized as they were viewed as low-grade professionals at the bottom of healthcare services – their low status frequently resulting in them being erased from the public’s mind eye. Recalling how her job was viewed by society prior to COVID-19, one participant states:

*“People didn’t really recognize us before. They see us at the bottom of healthcare. They think we just clean and wipe beds.” [Female, 31-40].*

Her account features a common characteristic among many participants: a bisecting of her work into a ‘before’ and ‘after’ the pandemic. Here, the participant spoke of the stigma of being reduced to ‘just’ performing gendered labour (wiping and cleaning beds), a sentiment echoed by in a subsequent account, wherein a participant discusses the theme of unrecognition/misrecognition within the context of health care professionals. Pinpointing nurses, she shares how these specific health care professionals forge differences between PSWs and their nursing profession by degrading (effectively, blemishing the character of) the former:

*“People don’t really think of us as having a good job. I’ve worked with nurses and they just see you as low grade. I don’t think they really appreciate what we do” [Female, 21-30].*

#### Different treatment due to background.

In addition to experiencing stigma from the general population and other healthcare professionals, immigrant PSWs specifically noted being stigmatized by Canadian-born PSWs. One participant recounted a negative remark that was made by a Canadian-born colleague:

*“‘You are from third world you should be happy of what you are earning here ‘” [Female, 41-50].*

While this account exemplifies the effort to use tribal stigma of ethno-national and racial identity to create differences within and across the PSW profession, it clearly reveals that immigrant PSWs experience marginalization both externally *and* laterally, from within their own professional occupation.

Furthermore, we found that among the immigrant PSWs, many had an education higher than was required to be a PSW. Despite the high educational background and training, immigrant PSWs contributions remained undervalued, as expressed by a participant who reiterated the lack of appreciation afforded to PSWs:

*“With this job you are not being appreciated. Whatever you bring from your background, no matter how smart you are, in this job you will not be accepted” [Female with a University Degree, 41-50].*

The frustration exhibited here was paralleled across several other participants accounts, their statements laced with ethno-national, gendered, and classed dimensions. Despite not using the words per se, participants were nonetheless sharing grievances with the ‘sticky floor’ – that is, the discriminatory (sexist, classist, *and* racist) socioeconomic conditions which keep working class, immigrant, and racialized women at the bottom of the employment scale, in low-skilled, low-paid, and deskilling jobs. Although the College of Nurses of Ontario (CNO) has improved the streamlining the process for internationally educated nurses to become licenced more quickly, immigrant PSWs expressed that they continue to face difficulties becoming licenced in their profession in Canada because they have been trained internationally [50]. This contributes to them staying fixed into low-skilled jobs, which, while necessary labour for the Canadian economy, are unsustainable for those seeking to compete and survive therein. The link between economic opportunity, ethno-national identity, and stigma was profoundly pronounced through one participants’ contention that:

*“As soon as they hear that accent, we are not given that opportunity. This is what the system has pushed us into” [Female, 41-50].*

In addition to being undervalued in the culture of the workplace and the system of the Canadian economy more broadly, immigrant PSWs described being treated unfairly through the process of client assignment. Relative to their Canadian colleagues, many noticed that they were being sent to care for clients with greater care demands in substandard living environments. In an undeniably reflexive discussion gender, class, race, and their sticky intersection, one participant reflectively shared:

*“Why are they sending Blacks? Why don’t they send their fellow white people? Because they know that we are coming from Africa, so they [the employer assumes] we are used to dirty things, so they are sending us to such places. We’re trying to take care of our own families, we’re trying to think of our children, we accept all kinds of jobs, jobs we are not supposed to accept” [Female from Nigeria, 41-50].*

This account, and the preceding accounts upon which it is set, all demonstrate the stigma that PSWs experience at the intersection of race, gender, class, and ethno-nationality. The ways in which these systems of subordination intersect was consistently evidenced through the stigma experienced – which was both moral/character and tribal in its materialization. Such stigmas were maintained at multiple scales –by clients, fellow Canadian-born PSWs, health care professionals, and the Canadian socioeconomic system at large. Despite such overwhelmingly negative experiences, there existed a myriad of novel and sometimes favourable experiences that were uniquely introduced by the pandemic. We turn our attention to these experiences now.

### PSWs perceptions during pandemic

#### Increased recognition/appreciation.

Once the pandemic began and PSWs together with other HCWs were labelled ‘essential’, the way they were viewed by society changed. Immigrant PSWs reported that they began feeling appreciated, as their role in the healthcare field was finally being recognized in the media, more importantly through the daily briefings by politicians and senior medical care staff. To show their appreciation, society began engaging in acts that were unheard of prior to the pandemic. One participant recalled how she felt about these positive actions:

*“I felt happy, I felt appreciated. When I drive around town and see people posting banners that say “Frontline workers we love you, Thank you” [Female, 41-50].*

Another participant elaborated on these positive responses by sharing a memorable experience she had:

*“On October 1* ^*st*^ *, the person on the radio complimented all the PSWs and all that we do. It felt so overwhelming. I felt so appreciated upon hearing it on the radio from someone, you know - I really never knew that I was so special. I know I am but to hear from someone there honestly, I got goose bumps just listening to the guy speak on the radio” [Female, 50+].*

A participant reflected on how those from other occupations such as police officers finally recognized PSWs’ importance in the healthcare system, once they were labelled as ‘essential’ workers:

*“If a policeman stops you, if you are an essential worker, he will allow you to go without asking questions. He knows that the people you are going to support, they need you during this time” [Male, 21-30].*

Even more minor and mundane actions began to occur once PSWs were labelled ‘essential’. Actions such as being thanked by community members began happening more frequently. A participant shared:

*“We are being recognized. People actually think that we are heroes. Especially since the start of* the *pandemic, when I go out to the store, people would say “Thank you for your service”. People are thanking me when they see me in scrubs and appreciate me. I cried, really, they didn’t notice us before and now in this pandemic, they appreciate us and they appreciate what we do” [Female, 41-50].*

Beyond the emotional effect of the labelling, immigrant PSWs greatly appreciated the wage increase from the Government of Ontario. Hence, participants also shared their disappointment that this wage increase was only temporary. A participant commented on this issue:

*“Personally, I thank the government for it. We appreciate it but we hope they will do more… but why is it only because of the pandemic they have to give us extra? You know, so if you know that [we’re important] why not continue paying?” [Female, 50+].*

When reflecting on their treatment prior to being labelled ‘essential’, participants expressed that they were frustrated that the impact of their work was not acknowledged or appreciated prior to the pandemic. Some participants mentioned that although their pre-pandemic duties and duties during the pandemic were similar, it was unfortunate that they were only seen as ‘essential’ once given this label. Certain populations such as the elderly, rely on PSWs’ work in order to carry out daily functions. A participant commented on how she felt about this:

*“The pandemic brought some kind of recognition, it made the government realize that these people have actually been very helpful because if they were not available, or if everybody decided to stay back at home, who would care for our seniors?” [Female, 31-40].*

#### Influence of the label ‘essential’.

Despite the adverse impacts of the pandemic, it proved very useful in highlighting the often under-valued work of immigrant PSWs to the general public especially. For many, these positive accounts reveal a particularly salient finding: these positive experiences of being referred to as essential provided a temporary buffer against the stigma of being a PSW.

For others, however, the label of ‘essential’ did little to change how they viewed themselves and their work. This was because they were already proud of the work they were doing, whether society considered their services ‘essential’ or not. A participant shared her thoughts:

*“I can’t say I’m proud of people calling me an ‘essential worker’ because I was proud of what I was doing before” [Female, 31-40].*

Although it appeared that society appreciated immigrant PSWs once they were labelled ‘essential’, some participants called it a ‘superficial reaction’ as the ‘essential’ label also drew some negative reactions from society. For example, some participants revealed that they felt as though society was afraid of them, viewing them as ‘dirty’, since they work in close contact with patients who had contracted the COVID-19 virus. On this, one participant recounted:

*“If you are even called an essential worker outside, there will be some people like very scared to get close to you, you know ‘he’s an essential worker don’t go to their house or don’t even work with them’” [Female, 31-40]*.

#### Public stigma.

Nevertheless, being labelled as ‘essential’ had a paradoxical effect, as it drew more attention to PSWs, socially attaching them to LTC facilities and their high reports of rapid and fatal COVID-19 outbreaks. This heightened visibility that immigrant PSWs faced quickly became problematic, as it led to their daily routines becoming more difficult and stressful. Under increased public and professional scrutiny, a regular task that could once be performed at ease was transformed stressful – their already spoiled identities further intensified by the taint of the virus. Speaking to this, a participant elucidated:

*“As an essential worker I have to dress differently. Normally, you could get dressed in your uniform and stop at the bank on your way to work. Now, you cannot do that because once people see you in your uniform they’re panicking” [Female, 41-50].*

The negative stigma that immigrant PSWs experienced seemed to be a common occurrence among other groups of essential frontline workers. Some participants shared that they witnessed several other essential workers being treated differently than the rest of society when performing daily tasks. A participant shared an occurrence she witnessed:

*“I witnessed an incident where a group of nurses were rejected at the front door of the bank because they were in their scrubs” [Female, 41-50].*

Additionally, immigrant PSWs faced stigmatization in an area that their Canadian-born co-workers did not, as they were being blamed for the transmission of the virus to Canada. A participant reflected on this experience:

*“At the beginning, it was us, the foreigners, that were been targeted - like it was us that brought it here [the virus to Canada]” [Female, 41-50].*

In light of the positive ascription of ‘essential,’ the quotations by the participants offer insight into some of the novel stigma negotiated by immigrant PSWs during the pandemic. Yet it in the process of doing their ‘essential’ work immigrant PSWs suffered negative experiences from employers. We will now delve into the workplaces experiences that immigrant PSWs encountered during the COVID-19 pandemic.

### Workplace experiences by PSWs

#### Undervalued.

Many news outlets posted clips of essential workers being praised and cheered on by community members. The headlines in the news even referred to ‘essential’ workers as ‘heroes’ [51]. Unfortunately, some participants in this study reported that employers do not treat them in this gratifying way. A participant expressed how she was treated by her employer during the pandemic:

*“It felt like I was a being called a superhero but not being treated as such” [Female, 21-30].*

#### Inadequate PPE.

Despite strict government protocols, unsafe working environments were a common concern raised by participants as some employers failed to provide their employees with sufficient personal protective equipment (PPE). This left many PSWs feeling unsafe at the workplace, as they were not able to carry out their duties safely. A participant shared her frustration at the very unorganized and delayed preparedness at her workplace:

*“They brought out only one shield and then said all of us are supposed to use it in turns… [my employer was] harassing me that the shield is supposed to be for all of us I said no. [the employer said] for all of you, you will you be sharing it. [When] people ask me “why do you have your own shield?” I said because I fought for it and I don’t think everybody should be using one shield” [Female, 41-50].*

Although some employers had a sufficient supply of PPE at the beginning of the pandemic, some still questioned staff about the use, implying that it should not be used as often. A participant described an interaction with her employer:

*“We were always short of PPE and they [employer] would complain that the sanitizer finished so quick... My employer would ask “how come the gloves finished so quickly?” I would say to them the main reason why it’s going faster now is because people are doing what they’re supposed to do” [Female, 41-50].*

#### Lack of respect from employers.

Beyond inadequate PPE, immigrant PSWs felt that they were not receiving enough support from their employers during this stressful period as their concerns were not being addressed. One participant expressed her frustration:

*“You [employer] are not listening to what we are trying to tell you, I’m telling you we need help... the management are not being supportive or listening to their staff” [Female, 21-30].*

Further, although inadequate employer support has been a longstanding issue in Canada, some respondents felt that this experience was heightened during the pandemic as the need for drastic operational changes at the workplace created avenues for perceived mistreatment and other related workplace complications. A participant shared that:

*“The work environment isn’t welcoming. There is a particular person that people complain about, the front desk person. They have a face mask on, which is understandable. But anything you touch, they don’t want to touch it anymore. If you’re filling out a form, they won’t even collect it from your hand, as if you have a problem” [Female, 31-40].*

#### PPE and communication challenges.

Immigrant PSWs also faced other barriers at the workplace when compared to their Canadian-born co-workers. For instance, immigrant PSWs found it difficult to communicate with their clients, as the mask made it more difficult to understand them because of their accent. A participant commented:

*“When you have the mask, talking to them [clients] is difficult. I can’t take off the mask to talk to them, since I am a person of color and my accent, I have to speak slowly” [Female, 50+].*

These experiences that participants shared demonstrate that employers were unprepared for a pandemic and were unsupportive, resulting in frustration for immigrant PSWs. Overall, the findings show the enduring stigma and challenges at the workplace during the pandemic that PSWs had to deal with while being labelled as ‘essential’ workers. The complex relationships between immigrant PSWs, their fellow workers and employers is reinforced by their vulnerabilities resulting in part from their immigrant status.

## Discussion

This study sought to assess immigrant PSWs experiences with and perceptions of being stigmatized yet labelled as ‘essential’ during the COVID-19 pandemic. Employing both Crenshaw [22] and Goffman’s [24] pioneering concepts, it sought to analyze the stigma negotiated by immigrant PSWs using an intersectional lens, so as to critically contemplate how the stigmatization they face is multifaceted. Such was evidenced across the presentation of findings, made clear through the fact immigrant PSWs experienced both Goffman’s [24] blemish of moral/individual character and tribal stigma in the workplace, pre- and peri-pandemic. Concerning the blemish of moral/individual character, this form of stigma was collectively ascribed onto the immigrant PSWs by: clients, Canadian-born PSWs, and nurses, who saw immigrant PSWs as ‘low-grade,’ ‘low-skilled,’ and differentiable from themselves. While this form of stigmatization predated the pandemic, it was intensified during this medical moment, as the blemish took on pathological character due to the virus. From their accounts, it is clear that blemishes of the character are inextricable from – and compounded by – the tribal stigma of race, ethno-national, gender, and class identities, for immigrant PSWs racialized bodies, ethno-national accents were continuously stigmatized by people both in and outside of the medical profession.

By exploring immigrant PSWs compounding and complex experiences of stigma, we were able to thread the needle between tribal stigma (racialization, ethno-national xenophobia) and individual character blemishes (classed, ‘low-skill’ professions), effectively illustrating how stigma is racialized, ethno-nationalist, and classed. The stigma that PSWs face is a result of these systems of domination intersecting on their bodies, in the workplace. In illustrating the relationship between intersectionality and stigma –essentially, that stigma is intersectional – this study identifies challenges often left out of political and media coverage, despite the fact that they constitute an important labor force in Canada.

Along with being associated with the original transmission of the virus, immigrant PSWs were often perceived as contaminated, carriers of the virus by society, due to potential exposure in the workplace. This resulted in them making changes to their daily routine, such as not being able to wear their scrubs in public when running errands, to avoid being noticed as a PSW. Furthermore, this perception resulted in society denying them access to necessary services such as the banks at the early stages of the pandemic. This is consistent with Smith et al.’s [30] argument that stigmatization occurs through both verbal or non-verbal forms that express the devaluation of the target, based on their actual or perceived membership in a stigmatized group.

Our findings are consistent with Taylor et al.’s [52], who reported that members of society in Canada and the United States did in fact stigmatize HCWs, by avoiding them for fear of contracting the virus, especially in heat of the pandemic. Our findings are also consistent with work by Hebl et al. [52], whereby immigrant PSWs believed that people were scared to come close to them when they were out in public, and people would panic when they saw the PSWs in their uniform. Thus, stigmatization can intensely skew social interactions [38]. Indeed, our findings demonstrate the social division created by stigma that Goffman has proposed, where society creates a division between ‘normal’ and ‘abnormals’, in this case it is non-HCWs who are viewed as ‘normals’ and the HCWs and immigrant PSW’s who are viewed as ‘abnormals’.

Some immigrants were treated poorly by their employers due to their immigrant status prior to the pandemic [53]. The persistent poor treatment of immigrant PSWs by their employers during the pandemic could be justified by Tyler’s [39] concept of ‘stigma power’, where stigma is a form of classificatory violence ‘from above’ which devalues people in the process of creating new opportunities for capital. This is worth noting given Canada’s neoliberal structured healthcare system, where power dynamics exist in relation to occupational hierarchies. Participants expressed that PSWs are often placed at the bottom of this hierarchy, lacking PPE and working in unsafe environments. In the context of this study, it could be said that participants experienced stigma in the form of classificatory violence ‘from above’, in this case, from their employers who are higher in the hierarchy, which led to them being devalued. The fact that some employers failed to give PSWs the support they asked for, such as sufficient PPE to allow them to carry out their duties safely emerged as a concern. It is thus evident that race, gender, class, and nationality were inextricably linked, fusing to create a unique type of stigma.

Not only did participants face stigmatization from employers, but also from other employees who were not working on the frontline. The stigmatization they faced from other employees made the workplace setting uncomfortable for them. This was particularly stressful when put in the context of a pandemic. This situation demonstrates the aspect of maintaining social power through stigmatization, whereby those with power stigmatize people with less power in order to maintain inequalities between groups. The result, a social division between the two groups, non-PSW co-workers who are considered ‘normal’ [24] and PSWs who are viewed as deviant due to the high-risk conditions they are exposed to. Consistent with Hebl et al. [38], stigmatization can reinforce tense social interactions between the stigmatized and non-stigmatized individuals, as demonstrated by the interactions that occurred at the workplace between PSWs and other types of employees.

Pre-pandemic, given that PSWs were stigmatized for being ‘low-skilled’ workers, they were not labelled as ‘essential’ and their work was largely unappreciated by society. Our findings revealed that once the pandemic began and they were labelled ‘essential’, their work gained traction in the general public. This was demonstrated by community members through their engagement in a range of positive acts of appreciation, including verbally thanking immigrant PSWs and displaying appreciative signs and murals. At the governmental level, PSWs were also given a temporary wage increase. However, once the high rates of COVID-19 began to drop, ‘essential’ workers were less often talked about in society and this wage increase was eventually removed. This demonstrates that labels and their related benefits are fluid and a relational process [40]. Specifically, our findings show the impact that the ‘essential’ label had on immigrant PSWs during the pandemic. Although labelling is often associated with negative consequences [42], this is an example where labelling partly affected a group positively.

Sjöström [42] suggests that labelling can sometimes be used as a way to exert control over a group. Consistent with Sjöström’s [42] argument, the labelling of PSWs and other frontline workers resulted in some form of control. For instance, this group was allowed to work, providing services to many individuals, while everyone else was on lockdown. Consistent with work by Pandey et al. [54] the ‘essential’ label is often given to those who are considered low-wage and low-status. Pandey et al. [54] highlight that labelling occupations as ‘essential’ is a way of devaluing paid domestic work and that this can be used as a control mechanism by employers to maximize labour.

Yet, making PSWs feel important was crucial in motivating them on the frontlines during the pandemic, especially considering there was already a shortage of PSWs [20]. In fact, the Government of Ontario states that the goals of the temporary pandemic pay increase was to encourage staff to continue working, to help maintain safe staffing levels and the operation of critical frontline services [5]. Consequently, the ‘essential’ label was used as a form of control in attempt to maximize labour during a time that PSWs services are crucial, especially given that the government is responsible for the funding of PSWs in LTC, home care, and community care, where the majority of PSWs in Ontario work [18].

Given that PSWs were already performing the same kinds of tasks pre-pandemic, we agree with Cox’s [55] assertion that labelling PSWs and HCWs as ‘heroes’ during the pandemic may have some unintended effects on these workers. For instance, Cox [55] argues that although some believe that the description healthcare ‘heroes’ may seem fitting, the narrative in the media may be unhelpful in various ways. For example, it could lead to some HCWs feeling that the level of personal risk they are currently being expected to accept at work is beyond what they ‘signed up’ to do. Thereby sending the wrong message to HCWs, that their work is too demanding and that they are expected to accept personal risk at work beyond what is expected of them; and if this is what is defined as ‘heroism’, we cannot expect HCWs to be ‘heroes’ long-term.

## Conclusion

This study contributes to literature of stigma and labelling in the context of health professions. First, participants’ statements merged to reveal that immigrant PSWs negotiated a complex and compounding form of stigmatization, owing to their location at the marginalized intersection of race, gender, class, and ethno-nationality. Just as their identities intersected, so too did the forms of stigma they encountered, as accounts’ spoke concertedly of both tribal (racial, ethno-nationality) and individual/moral stigma (working in low-skilled labour) in the workplace. Furthermore, findings show that immigrant PSWs, from the onset of the pandemic faced several challenges, such as initially being stigmatized as potential COVID-19 spreaders, while working with dysfunctional PPE, in unsafe work environments. Yet, the political labelling of PSWs and HCWs as ‘essential’ workers brought both a positive appeal for the recognition received, but also with an underlying meaning of control. Moving forward, it is important that PSWs’ wage is reconsidered, as the pandemic has shown the intensity of their work and their efforts should not go unnoticed and unappreciated. By increasing the wage of PSWs, this could help retain them in the workforce, supporting the sustainability in addressing the ongoing shortage of PSWs.

To help prevent a further shortage of PSWs, it is important to address the workplace challenges faced by PSWs. Some of the workplace challenges that were intensified during the pandemic, including unsafe working conditions and employers disregarding PSWs’ concerns. These issues may have been addressed if a regulatory body existed for PSWs. It emerged from this study that PSWs faced many difficulties related to not having a regulatory body prior to the pandemic, and the pandemic has further demonstrated the need for this [56]. One issue that could be addressed if a regulatory body existed for PSWs is the enhancement of safety regulations to better protect PSWs in the workplace, particularly regarding emergency preparedness. For example, implementing a regulation that requires workplaces to maintain an emergency supply of PPE would contribute to ensuring that every worker has adequate protection in the case of another pandemic. This would help address concerns related to having to share PPE that were raised by PSWs. Another issue that could be addressed with a regulatory body would be improving worker protection and rights. By doing this, employers will have to address PSWs’ concerns instead of disregarding them which PSWs’ expressed was an issue during the COVID-19 pandemic.

In terms of the stigma that PSWs face, it is important that there is increased awareness surrounding the impacts that stigmatization has on the psychological and physical health of PSWs, as stigma positively impacts fatigue and burnout, while negatively impacting satisfaction [57]. If PSWs continue to face high levels of stigmatization, it is expected that there will be at minimum a temporary loss of employees in these fields resulting from PSWs needing time for their recovery from the health issues that they will face. Furthermore, high levels of stigma would counteract the perceived benefit of labelling PSWs as ‘essential’ in order to retain PSWs in the workforce through motivating them if this label leads to stigmatization. This could ultimately lead to a range of negative health outcomes, leading back to the original problem of a further shortage of PSWs. Relative to the stigma related to fear PSWs’ transmitting COVID-19, evidence has indicated that HCWs’ clothing is often contaminated by microorganisms or pathogens that can cause infections or illnesses [58]. PSWs may help reduce the stigma they often encounter while also reducing the chances of transmitting illnesses if they do not wear their uniforms outside of the workplace. Given this, a policy consideration regarding uniforms is for employers to strengthen enforcement of the practice of not wearing uniforms outside of the workplace. This may require employers to offer a dedicated space for staff to change before starting and after completing their shifts.

Overall, the COVID-19 crisis has demonstrated that immigrant PSWs faced many challenges in relation to being labelled, stigmatized, and their employer and workplace. We have proposed that many of these issues have stemmed from the use of “stigma power” by employers [39]. Informed by the frameworks of stigma and intersectionality, this study has helped shed light on the issues that remain in this field, even after PSWs have experienced advantages associated with being labelled ‘essential’; such as being recognized and appreciated in their job. It is important that we continue to give them the recognition that they deserve, as they have indicated that they truly value appreciation from society. Furthermore, it is crucial that the issues that were highlighted by these experiences be addressed, as PSWs play a vital role in our healthcare system and their importance will only increase as our population continues to age [15].

## Study limitations

Since the participants in this study were only from Windsor, Ontario, this gives rise to the potential of external validity bias as the challenges the participants faced may not reflect all the challenges that immigrant PSWs faced during the pandemic who are from other areas of Ontario [59]. The interpretive validity of this study was limited by language barriers, as the first language of participants in the study was not English, and the interviews were conducted in English. The language barrier has a potential to influence participants’ ability to effectively explain their experiences and perceptions, limiting the overall quality of their answers. The overall quality of responses generated from the interviews has limitations that can be attributed to participants’ misinterpreting questions if language barriers limited their understanding of the questions being asked. Additionally, the language barrier may have led to participants using the wrong choice of words when answering questions, leading to unclear or distorted messages, which could lead to the findings being misinterpreted [60]. Since a translator or interpreter was not present during the interviews to reduce the likelihood of misinterpretation, there was a greater risk related to misinterpretations of participants’ responses [61].
